# The association between quality of life(QOL) and health literacy among junior middle school students: a cross-sectional study

**DOI:** 10.1186/s12889-018-6082-5

**Published:** 2018-10-19

**Authors:** Min Ran, Linli Peng, Qin Liu, Michelle Pender, Fang He, Hong Wang

**Affiliations:** 1 School of Public Health and Management, Chongqing Medical University, Research Center for Medicine and Social Development, Collaborative Innovation Center of Social Risks Governance in Health, Chongqing Medical University, Chongqing, 400016 China; 2 Duke Global Health Institute, Duke University, Durham, NC 27708 USA

**Keywords:** Junior middle school students-health literacy-quality of life(QOL)-cross-sectional study

## Abstract

**Background:**

Lower health literacy is associated with poor quality of life (QOL) among patients with chronic disease; little is known about this relationship among the general population, especially for child and adolescent. To fill this gap, this paper aimed to investigate the association between health literacy and QOL in junior middle school students, and explore how QOL varies by health literacy.

**Methods:**

An anonymous cross-sectional survey was conducted among junior middle school students (aged 12–15) from Shapingba district, Chongqing in China, and participants were recruited using stratified cluster sampling. Health literacy and QOL were measured using two validated scales, and quantified using a five-point Likert scale with health literacy classified as low, medium, or high. We used multivariable logistic regression to test adjusted association between health literacy and QOL.

**Results:**

A total of 1774 junior middle school students were evaluated, with the mean age was 13.8 ± 1.0 and of whom 905 (51.0%) were male. About 25.5% of the research subjects had a low health literacy. When controlling for age, grade, family structure and other covariates, highest discrimination was found among participants with low to high health literacy. Overall, Students who equipped with higher health literacy was associated with greater QOL (*P* < 0.01), and this discrimination remained significant in subscales: physiological well-being (*P* < 0.01), mental well-being (*P* < 0.01), social well-being (*P* < 0.01) and pubertal well-being (*P* < 0.01).

**Conclusions:**

The prevalence of low health literacy among junior middle school students in Chongqing area was relatively high, and inadequate health literacy may contribute to poorer QOL among junior middle school students. It merits further longitudinal studies to confirm the impact of health literacy on QOL. Overall, to improve students’ QOL, public health efforts for further improving awareness and enhancing effective promotion and education are urgently needed in junior middle school students, especially for low health literacy populations.

## Background

Despite the lack of uniform standard for the definition of QOL, there is a shared understanding to regard the concept of QOL as “the individual’s perception of their position in life in the context of the culture and value systems in which they live, and in relation to their goals, expectations, standards, and concerns” [1]. The level and determinants of QOL in adults have been well investigated in developed countries such as the US [2], the UK [3], Netherlands [4], and Sweden [5]. However, few studies have been reported on QOL in developing countries, especially for children and adolescents. Junior school stage is a necessary period of transition from childhood to maturity, which is critical for individual physical, psychological and social adaptation. During this period, the physical and psychological health and QOL may affect their life, so it is of far-reaching significance to study the QOL of junior school students. Since the 1950s, studies on QOL have been carried out in the United States, Canada, Western Europe, Eastern Europe and some Asian countries, while studies on QOL in China started late in the mid-1980s, and research on QOL in children and adolescents starts even later [6]. Xiao Cailan et al.’s research on primary and middle school students in Hebei province China found that students with a QOL above the middle level accounted for 67%, and there was still much room for improvement in the QOL of middle school students [6]. This result is lower than Wang’ research on children and adolescents in Chongqing, which found nearly 80% of students with a QOL above the middle level [7]. In short, existing researches have shown the QOL of children and adolescents at home and abroad is not high. In addition, those researches that do exist managing QOL among children and adolescents mainly focused on various pediatric diseases [8–12]. Researches on QOL amongst community populations of children and adolescents are needed. In our previous cross-sectional study of QOL among junior middle school students, we found that QOL may be influenced by demographic factors (e.g. sex, parent education level, family structure, parenting), the presence of chronic disease and poor vision [13]. Additionally, QOL may be in the position of affecting students’ daily performance, activities, and communication.

Health literacy, “degree to which individuals have the capacity to obtain, process, and understand basic health information and services needed to make appropriate health decisions” [14], has become an important health policy and health promotion agenda item in recent years. Unlike fixed social structures and demographic characteristics, which cannot be altered without massive social changing and political action, health literacy is susceptible to social environments and can be improved by targeted health education [15]. Although epidemiological estimate on the level of health literacy varies depending upon the characteristics of the sample, the measurement methods, standardized assessment means, and heterogeneity in the source of information, studies indicate that the health literacy of Chinese children and adolescents was generally low [16–20]. Data from a nationwide survey on health literacy showed merely 25.2% of adolescents aged 15–24 years with adequate health literacy skills, 15.9% with adequate knowledge, and 6.4% with adequate behaviors [21]. In another study in Guangdong, Xiao-Hua Ye et al. found the prevalence of adequate health literacy among in-school adolescents was only 14.4% [22], which was much lower than the 52% found in high school students in Texas US [23] and 41% found in Taiwan area [24]. A rigorous body of work in the previous day have established a clear link between inadequate health literacy and a wide variety of negative health outcomes in adults, including increased hospitalizations [25], greater use of emergency care [26] and poorer health status [27]. Fewer studies, however, are available in children and adolescents, and those that do exist demonstrate an association between lower parent or caregiver health literacy and more dosing errors in pediatric medication [28], worse child asthma and diabetes care [29, 30], lower child health insurance coverage [31], and higher pediatric emergency department use and outpatient visits [32]. Indeed, the decisions of parents or caregivers do impact children’s health status, however, children can also be in a position to decide their own health.

Evidence [33–35] continues to grow linking limited health literacy to poor QOL among patients in the medical/clinical context, however, whether health literacy significantly influences QOL among general populations of students has been seldom reported. To address this need, we explored the relationship between QOL and different levels of health literacy among junior middle school students, while controlling for other covariates (e.g. sex, grade, place of residence, economic status, parent education attainment, and so on).

## Methods

### Design and setting

A multistage cluster sampling design was used to select four junior middle schools in Shapingba District, Chongqing, China. Data were collected between November and December 2016. First, two urban junior middle schools and two rural junior middle schools were randomly selected from the district. Then, three to five classes were chosen randomly from grade 7 to 9 in the selected four schools. Lastly, all the students in the selected classes participated in the study. A cross-sectional survey was conducted with the chosen 1832 junior middle school students, of them 1774 students completed a 40-min survey (Mean = 13.83 years old; SD = 1.06) without apparent logical errors or missing items, yielding a response rate of 96.83% (1774 of 1832). The study was approved by Biomedical Ethics Committee of Peking University (IRB 00001052–13,034) and the ethical committee of the Chongqing Medical University, and written informed consent was obtained from students and their parents before their participation in the study.

### Measurements

1. Socio-demographic Information

A self-administered questionnaire was developed to collect the following information: age, sex (male/female), grade, whether a only one child (yes or no), place of residence ((1) urban: capital cities or county capitals;(2) rural: capital city suburbs, towns, or rural villages), educational attainment of parents((1) low: illiteracy, primary, or junior middle school; (2) medium: senior middle school or technical secondary school; and (3) high: college or higher), family economic status (poor, medium, high), family relationship (harmonious, neutral, poor), and the students’ perception of school achievement (fair, moderate, bad). These characteristics were included as covariates in data analyses.

2) Health literacy

The health literacy scale developed by Wang Lingyi et al. [36] is a 50-item instrument designed to assess the subjective health literacy of middle school students. It based on the definition of health literacy provided by the World Health Organization (WHO) [37], “Notice of the China’s ministry of education on the issuance of the Outline of health education guidance in primary and secondary school students” [38] and “Chinese citizens’ health literacy-basic knowledge and skills (Trial)” [39]. The 50 items are rated on a five-point Likert scale (1–5 points), and minimum to maximum possible scores to be obtained from the scale could range from 50 to 250 points. According to the score, health literacy was categorized into 3 levels of performance, using quartiles as the cut-off points: low literacy (scores of </= 183,1st quartile), medium literacy (scores of > 183 and < 210, 2nd quartile), and high literacy (scores of > = 210, 3rd and 4th quartile). A high score indicated that the student’s health literacy level is high. In the original study, the Cronbach’s α was 0.948, and two-week test-retest reliability was 0.840. The validity analysis (*χ*^*2*^ = 11,250.24,*p* < 0.0001, Tucker-Lewis index (TLI) = 0.92, comparative fit index (CFI) = 0.92, adjusted goodness of fit index (AGFI) =0.50, root mean square error of approximation (RMSEA) =0.088) confirmed that the scale consisted of 3 core dimensions, which refer to functionality, interactivity and critical evaluation of health literacy. In this present study, the Cronbach’s alpha value indicated the reliability of the internal consistency at 0.96, the result resembled that from our previous study [36].

3) Quality of life

QOL for children in puberty is a version of assessment to measure the QOL among the general population of children in puberty. It was constructed from three validated measures of QOL, including Inventory of Subjective Life Quality for Child and Adolescent (ISLQ) [40], Child and Adolescents Quality of Life Scale (CAQOL) [41], and Chinese Version of Peds QL4.0 [42]. The forms consist of 39 items distributed into the following 4 subscales: physiological (8 items), mental (11 items), social (14 items) and pubertal well-being (6 items).Each item addresses the student’s experiences over the past three months and is rated on a 5-point Likert scale (5 = never,1 = always) with item 23 scores reversed. Person’s mean score from the questions within each category is used to calculate the subscales and total scale score, with higher scores demonstrating better QOL. Correlation with the comparable QOL scale (ISLQ) [40] has shown an acceptable convergent validity as the correlation coefficient between the two scales is 0.76. The validity (*χ*^*2*^/df = 4.24, CFI = 0.95, NFI = 0.94, NNFI = 0.92, GFI = 0.96, AGFI = 0.92, RMSEA = 0.08) and reliability (Cronbach’*α* coefficient was 0.89 for total scale and 0.64–0.85 for four dimensions; two-week test-retest coefficients are good to excellent, with ICC from 0.72 to 0.88) of this scale has established in the original research. As for this present study, the measurement has shown good reliability, with the Cronbach’s alpha of the total scale was 0.89, and 0.82, 0.76, 0.88 and 0.65 for each subscale, respectively.

### Statistical analyses

Data analyses were conducted using the SAS statistical software package (SAS Institute, Cary, NC; version 9.2). Equality of means among characteristics groups were examined using t-tests or F-tests. For the ease of analysis, health literacy was categorized into a ternary variable of three categories, the low, moderate, and high levels. Chi-squared tests, t-tests or F tests were employed to examine the unadjusted associations between the health literacy level and participants characteristics. Further multivariate analyses were conducted using two sets of regression analyses, to examine whether QOL scores were associated with health literacy, without controlling for covariates, and then after controlling for covariates. Five separate analyses were conducted for overall and each of the four domain-specific QOL scores. In regression analyses, all significant variables identified in the univariate analyses were included and all observations missing on any variables were excluded from the analysis. Any result with a *P*-value less than 0.05 was considered statistically significant.

### Quality control

We piloted the survey instrument to identify any problems which might occur during the test, and checked the scale for reliability and validity. We implemented the survey with permission from the Headmaster, and the survey was administered in each class by trained investigators, and each student completed their survey independently. All questionnaires were issued immediately before students completed them, and were then reviewed by the investigators to ensure students had completed the forms correctly immediately after completion.

## Results

### Subject characteristics

A total of 1774 junior middle school students completed the survey, with the mean age was 13.8 ± 1.0. Of whom 905 (51.0%) were male, 564 (31.8%) were in the seventh grade, 878 (49.5%) lived in rural areas, 853(48.1%) were only children. As for the students’ fathers and mothers, 1128 (63.6%) and 1149 (64.8%) of them had junior middle school or below degree. Of the students, 1260 (71.0%) came from a household with medium economic status, 1004 (56.6%) had a harmonious family relationship, and 711 (40.1%) considered their school achievement as bad. Students’ characteristics by health literacy level are shown in Table 1. The mean score of health literacy was 194.11 (SD = 27.32), and about 25.5% of the research subjects had a low health literacy. T-tests or F tests revealed that low health literacy was more prevalent among males (*P*^a^ = 0.048), higher grade (*P*^a^ = 0.021), residents of rural areas (*P*^a^ < 0.0001), those whose father or mother were with less education (*P*^a^ < 0.001 or *P*^a^ = 0.021), those with poor family relationships (*P*^a^ = 0.017) and poor economic status (*P*^a^ = 0.010), and those who considered their school achievement as bad (*P*^a^ = 0.017). Chi-square tests showed the similar results.

**Table 1:** Characteristics of junior middle school students as a group and stratified by health literacy level

Characteristics	The score of health literacy X(SD)	*P*^a^-value	Health literacy level *N*(%)			*P*^b^-value
			low	medium	High	
Sex, *N*(%)						
Male, 905 (51.0)	193.50 (28.54)	0.048	255 (28.2)	403 (44.5)	247 (27.3)	0.004
Female, 869 (49.0)	194.64 (26.00)		198 (22.8)	453 (52.1)	218 (25.1)	
Grade, N(%)						
7th,564 (31.8)	195.70 (26.65)	0.021	139 (24.6)	257 (45.6)	168 (29.8)	0.045
8th,611 (34.4)	195.09 (26.04)		143 (23.4)	313 (51.2)	155 (25.4)	
9th,599 (33.8)	191.60 (29.04)		171 (28.5)	286 (47.7)	142 (23.7)	
Place of residence, *N*(%)						
Rural,878 (49.5)	190.40 (25.15)	< 0.0001	272 (31.0)	441 (50.2)	165 (18.8)	< 0.0001
Urban,896 (50.5)	197.73 (28.86)		181 (20.2)	415 (46.3)	300 (33.5)	
Only child, N(%)						
Yes,853 (48.1)	194.69 (28.35)	0.383	210 (24.6)	399 (46.8)	244 (28.6)	0.088
No,921 (51.9)	193.56 (26.34)		243 (26.4)	457 (49.6)	221 (24.0)	
Father’s education level, *N*(%)						
Low,1128 (63.6),	192.99 (26.65)	0.001	309 (27.4)	559 (49.6)	260 (23.0)	< 0.0001
Medium,471 (26.6)	193.99 (31.00)		117 (24.8)	214 (45.4)	140 (29.7)	
High,175 (9.9)	201.60 (26.15)		27 (15.4)	73 (47.4)	65 (37.1)	
Mother education level, *N*(%)						
Low,1149 (64.8)	192.87 (25.32)	0.021	323 (28.1)	564 (49.1)	262 (22.8)	< 0.0001
Medium,484 (27.3)	195.75 (30.23)		111 (22.9)	225 (46.5)	148 (30.6)	
High,141 (7.9)	198.51 (31.69)		19 (13.5)	67 (47.5)	55 (39.0)	
Economic status, N(%)						
Poor,249 (14.0)	190.04 (27.75)	0.010	85 (34.1)	110 (44.2)	54 (21.7)	0.002
Medium,1260 (71.0)	194.23 (26.78)		314 (24.9)	620 (49.2)	326 (25.9)	
Good,265 (14.9)	197.33 (29.08)		54 (20.4)	126 (47.5)	85 (32.1)	
Family relationship, N(%)						
Harmonious,1004 (56.6)	196.59 (28.47)	0.017	216 (21.5)	475 (47.3)	313 (31.2)	< 0.0001
Neutral,688 (38.8)	190.74 (24.85)		212 (30.8)	347 (50.4)	129 (18.8)	
Poor,82 (4.6)	191.90 (29.81)		25 (30.5)	34 (41.5)	23 (28.0)	
Perception of school achievement, *N*(%)						
Fair,503 (28.4)	197.41 (28.83)	< 0.0001	98 (19.5)	238 (47.3)	167 (33.2)	< 0.0001
Medium,560 (31.6)	196.43 (25.78)		123 (22.0)	279 (49.8)	158 (28.2)	
Bad,711 (40.1)	189.94 (27.32)		232 (32.6)	339 (47.7)	140 (19.7)	
Total, 1774 (100.0)	194.11 (27.32)		453 (25.5)	856 (48.3)	465 (26.2)	

P^a^ Health literacy as a continuous variable, *P*^a^ value was based on t-test or F test

P^b^ Health literacy as a categorical variable, *P*^a^ value was based on chi-square test

**Fig. 1:**
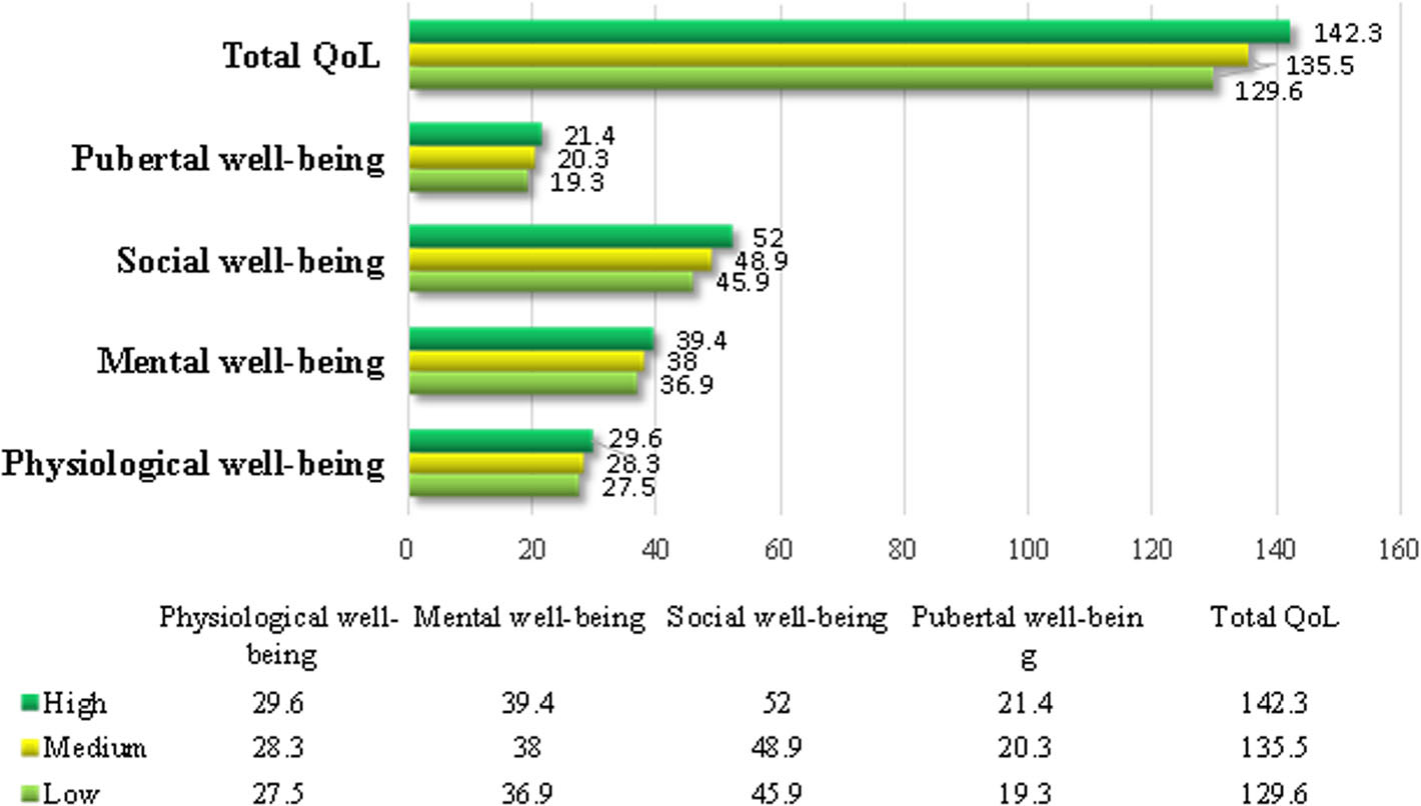
The scores of overall and each subscale QOL grouped by health literacy levels among junior middle school students (*N* = 1772)

The mean score of QOL among junior middle school students was 135.7 (SD = 17.4), and the total QOL score of students with high health literacy was significantly higher than those with medium (142.3 vs 135.5, *p* < 0.0001) or low health literacy (142.3 vs 129.6, *p* < 0.0001). The results for each subscale were similar (Fig. 1).

### Association between health literacy and overall QOL

As the unadjusted linear regression model (Model 1 in Table 2) showed, the QOL score was lower in the low health literacy (b = − 12.7, *P* < 0.0001) and medium health literacy (b = − 6.8, *P* < 0.0001) group as compared to the high health literacy group, that is, participants with higher health literacy reported better QOL. After accounting for covariates (Model 2 in Table 2), the associations remained significant between the high health literacy group and medium (b = − 5.0, *P* < 0.0001) or low (b = − 9.3, *P* < 0.0001) health literacy group. What’s more, the low health literacy group reported a somewhat larger decrement in the QOL scores than the medium health literacy group (− 12.7 vs − 6.8 in the unadjusted model; − 9.3 vs − 5.0 in the adjusted model) when compared to the high health literacy group.

**Table 2:** Standardized Coefficients Obtained From Multiple Regression Analyses Predicting QOL Scores from Health Literacy Levels and Characteristics without and with Controlling for Covariate

Independent Variable(s)	Unadjusted Model^a^(*N* = 1774)		Fully adjusted Model^b^(*N* = 1774)	
	b(SE)	*P*-Value	b(SE)	*P*-Value
Health literacy levels				
low	−12.7 (1.1)	< 0.0001	−9.3 (1.1)	< 0.0001
Medium	−6.8 (1.0)	< 0.0001	−5.0 (0.9)	< 0.0001
high	Ref.		Ref.	
Sex				
Male	Ref.		Ref.	
Female	−3.5 (0.8)	< 0.0001	−4.5 (0.7)	< 0.0001
Grade				
7th	Ref.		Ref.	
8th	1.3 (1.0)	0.1845	1.6 (0.9)	0.0766
9th	−3.0 (1.0)	0.0035	−1.8 (0.9)	0.0494
Place of residence				
Rural	Ref.		Ref.	
Urban	2.8 (0.8)	0.0007	1.8 (0.9)	0.0494
Only child				
Yes	Ref.		Ref.	
No	0.6 (0.8)	0.4630	−0.1 (0.8)	0.9349
Father’s education level				
Low	Ref.		Ref.	
Medium	2.8 (0.9)	0.0037	0.7 (0.9)	0.4498
High	4.2 (1.4)	0.0027	−0.4 (1.5)	0.8149
Mother’s education level				
Low	Ref.		Ref.	
Medium	4.1 (0.9)	< 0.0001	2.2 (0.9)	0.0184
High	4.2 (1.5)	0.0069	2.2 (1.6)	0.1868
Economic status				
Poor	Ref.		Ref.	
Medium	8.6 (1.2)	< 0.0001	4.2 (1.1)	< 0.0001
Good	12.5 (1.5)	< 0.0001	6.0 (1.4)	0.0001
Family relationship				
Poor	Ref.		Ref.	
Neutral l	10.8 (1.9)	< 0.0001	9.9 (1.8)	< 0.0001
Harmonious	21.7 (1.8)	< 0.0001	18.8 (1.8)	< 0.0001
Perception of school achievement				
Bad	Ref.		Ref.	
Medium	6.2 (1.0)	< 0.0001	4.0 (0.9)	< 0.0001
Fair	7.5 (1.0)	< 0.0001	4.2 (0.9)	< 0.0001

^a^ Unadjusted model: multiple linear regression models with QOL scores as the dependent variable, health literacy levels as the independent variable, adjusting for no characteristic

^b^ Fully adjusted model: adjusting for all covariates, including sex, grade, place of residence, whether an only child, parents’ education level, household income, family relationship, student’s perception of school achievement, and health literacy levels

Regarding to the characteristics, QOL scores were significantly higher among the students living in urban areas (b = 2.8, *P* = 0.0007), whose parents’ education level were relative higher, with medium (b = 8.6, *P* < 0.0001) or good (b = 12.5, *P* < 0.0001) house income, with neutral (b = 10.8, *P* < 0.0001) or harmonious (b = 21.7, *P* < 0.0001) family relationship, and with medium (b = 6.2, *P* < 0.0001) and fair (b = 7.5, *P* < 0.0001) consideration of their school achievement in the unadjusted model. Similarly, after further including covariates into the model, the discrepancy still remained in the groups of sex, grade, residence, economic status, family relationship, and perception of school achievement.

### Association between health literacy and domain-specific QOL

Covariates which were statistically significant in the fully adjusted model were chosen to enter the multivariate linear regression analysis. Results showed in Table 3 indicated that, compared to the students with high health literacy, students in the low health literacy group had significantly lower QOL scores in overall (b = − 9.8, *P* < .0001), physiological-specific domain (b = − 1.7, *P* = 0.0032), mental-specific domain(b = − 2.3, *P* = 0.0028), social-specific domain (b = − 4.1, *P* < 0.0001) and pubertal-specific domain (b = − 1.7, *P* < 0.0001) scales. For research subjects in the medium health literacy group, the results were similar. Furthermore, coefficients estimated for the low health literacy group were consistently stronger than those estimated for the medium health literacy group (− 9.8 vs − 5.1,-1.7 vs − 1.0, − 2.3 vs − 1.1, − 4.1 vs − 2.0, − 1.7 vs − 0.9), situation was same both in overall scale and specific domain subscale. That is, students with lower health literacy were associated with lower physiological-specific, mental-specific, social-specific, pubertal-specific and total QOL. Beyond that, the dependent variable that most strongly affected by the students’ health literacy were Social well-being-specific QOL (b = − 4.1), which was the largest contributor to the whole effects modified by health literacy levels (Table 3).

**Table 3:** Multivariate linear regression models predicting QOL (total and subscale) scores for the entire sample

HL levels	QOL total score		Physiological well-being		Mental well-being		Social well-being		Pubertal well-being	
	b(SE)	*P*-Value	b(SE)	*P*-Value	b(SE)	*P*-Value	b(SE)	*P*-Value	b(SE)	*P*-Value
Low	−9.8 (1.9)	< 0.0001	−1.7 (0.6)	0.0032	−2.3 (0.8)	0.0028	−4.1 (0.8)	< 0.0001	−1.7 (0.4)	< 0.0001
Medium	−5.1 (1.1)	< 0.0001	−1.0 (0.3)	0.0028	−1.1 (0.5)	0.0188	−2.0 (0.5)	< 0.0001	−0.9 (0.2)	< 0.0001
High	Ref.		Ref.		Ref.		Ref.		Ref.	

*HL* The short test of health literacy, *QOL* The short test of quality of life

Multiple linear regression models with QOL scores as the dependent variable, health literacy levels as the independent variable, and sex, grade, place of residence, mother’s education level, household income, family relationship, and student’s perception of school achievement as covariates

## Discussion

The impacts of health literacy on health status and health outcomes have been widely discussed in the previous literature [27, 28]. Nevertheless, to our knowledge, this is the first study to examine the relationship between health literacy and QOL in general populations of junior middle school students. In concordance with our hypothesis, health literacy emerged as a moderator for the QOL in junior middle school students. Results indicated that students with low health literacy had a higher risk to get fewer scores of QOL, even adjusting for social-demographics. Furthermore, the associations between health literacy levels and QOL of overall and each subscale were more pronounced in the low health literacy group than in the medium health literacy group.

After categorizing the health literacy into 3 levels by using quartiles as the cut-off points, we found that about one-quarter of students presented low health literacy,which is higher than other studies reported with samples from different age groups and using different previously validated evaluation instruments, such as Chang and her colleagues’ study [43] measured by the Chinese version of Test of Functional Health Literacy (TOFHL)for adolescents among Taiwanese students, and the results reported by Chisolm et al. [44] assessed with the Rapid Estimate of Adult Literacy in Medicine, teen version (REALM-Teen). Due to the lack of studies specifically conducted among teenagers, together with the scale we used in this present study differs from the other’s, little information can be compared on the overall health literacy level of teenagers in different regions, not to mention any regional comparisons.

Moreover, we found that students with low health literacy were more likely to get fewer scores of QOL comparing to their counterparts with high HL, even adjusting for covariates. This finding amongst junior middle school students is concordance with reported findings among adults and patients [35, 45, 46]. For instance; a study among patients with ischemic heart disease showed that increasing health literacy may improve health-related QOL and reduce the impact of ischemic heart disease [35]. In another study, Cuili Wang and her colleagues found that low health literacy was associated with poorer health-related QOL in the context of chronic disease [45]. However, owing to the lack of study addressing the relationship between health literacy and perception of QOL by the general adolescent public, comparisons of results in this age group would be difficult. There are several reasons may explain why junior middle school students with high health literacy levels have a higher QOL than those with low health literacy levels. Firstly, students with higher health literacy levels may be more prone to expose to the health-related information, which provided by medical professionals, parents, teachers and friends. Once equipped with a solid understanding of their health, these students are more likely to make better decisions to improve their QOL. Instead, poor health literacy may limit students’ ability to communicate with health providers, understand and follow health providers’ instructions, thus, leaving them without the appropriate knowledge to make informed decisions [47]. Secondly, since numerous studies [29–32] have demonstrated the impact of parent/caregiver’s health literacy on the health status or outcomes of their children, together with children’s health literacy are significantly associated with their parent/caregiver’s health literacy [48], there is every reason to believe children can also be in a position to decide their own health and QOL. Lastly, students with low health literacy are more likely to engage in risky behaviors such as smoking, alcohol consumption, insufficient physical activity, unhealthy dietary intakes. All these behaviors have been identified significantly associating with poor QOL [44]. As for the specific domains of QOL, our results corroborate the Ownby RL study [49] which found that those with low health literacy experience poor QOL and suffer from more mental and physical health problems. In addition, our findings showed that low health literacy was associated with low pubertal well-being.This can be partly explained as junior middle school students are right in their physical and psychological development stage, they are prone to come up with physical or psychological problems.

The association between low health literacy and QOL could be recognized in a wider context of the influencing factors of health literacy. Data analysis showed that, students living in urban areas were more likely to have higher health literacy than their urban counterparts, consistent with our expected and other studies in China [50], which may be related with the degree of economic development, the disequilibrium of health-related resources and the accessibility of health information [50, 51]. Regarding to the discrepancy of sex, most of the studies [46, 52] point to better health literacy for females, that corroborates our findings. There were several possible explanations for this divergence. For one thing, female students were more concerned about health information and were more willing to turn to families, friends, teachers and mass media for health-related information [53]. For another, female students may pay more attention to personal image and health-related details than males. Additionally, we found that students who attained a low health literacy level were more likely to come from a low familial resource environment, such as low income, low parental educational levels, and poor family relationship. It would be inherent to associate a low familial resource environment with low household economic status, lower parental educational levels, higher proportion of manual labour employment, high prevalence of parental unhealthy behaviors, and poorer dietary pattern. All these factors involved have been identified as the risky factors for low QOL. Similar results were found in earlier studies [54–56].

### Limitations

This study represents exploratory work in a new area, and as such, there are several limitations that should be considered in the interpretation of the results. First, we examined a population of junior middle school students from four selected junior middle schools in one district in Chongqing which may not represent the whole population of junior middle school students. Secondly, the study was a cross-sectional study and causality cannot be established with this study design. Finally, health literacy and QOL were measured using self-report surveys, and confounding factors could have influenced the students’ responses. Further studies are necessary to better establish whether adequate health literacy results in improving QOL, and the extent to which it is improved. Additionally, the sample should be increased, and students from different districts should be added as a comparison in order to strengthen the findings of future studies.

## Conclusions and implications

This study suggested that the inadequate health literacy may contribute to poorer QOL among junior middle school students. Improving health literacy among students may have a positive effect on their QOL. Therefore, the study highlights the importance of strengthening efforts to increase the level of health literacy across adolescents and teenagers, especially among those from a low familial resource environment and those living in rural areas. Overall, to improve students’ QOL, public health efforts for further improving awareness and enhancing effective promotion and education are urgently needed in junior middle school students, especially in low health literacy populations. Additionally, it merits further longitudinal studies to confirm the impact of health literacy on QOL, and verify the hypothesis that improving students health literacy may be effective in modifying QOL.

## References

[1] World Health Organization (1995). The World Health Organization quality of life assessment (WHOQOL): position paper from the World Health Organization. Soc Sci Med.

[2] Nyman JA, Barleen NA, Dowd BE (2007). Quality-of-life weights for the US population: self-reported health status and priority health conditions, by demographic characteristics. Med Care.

[3] Lumley S, Ward P, Roberts L (2015). Self-reported extracurricular activity, academic success, and quality of life in UK medical students. Int J Med Educ.

[4] De Rooij AH, Luijkx KG, Declercq AG (2011). Quality of life of residents with dementia in long-term care settings in the Netherlands and Belgium: design of a longitudinal comparative study in traditional nursing homes and small-scale living facilities. BMC Geriatr.

[5] Bingefors K, Lindberg M, Isacson D (2011). Quality of life, use of topical medications and socio-economic data in hand eczema: a Swedish nationwide survey. Acta Derm Venereol.

[6] Xiao Cailan. Study on the quality of life and influencing factors of middle school students[D]. Hebei Medical University, 2012. 10.7666/d.y2105196.

[7] Hong W, Yi Z, Dawei L (2007). Analysis of quality of life and its influencing factors of junior high school students in Chongqing. Modern Preventive Medicine.

[8] Van GR, van Essen LE, Rovers MM (2007). Quality of life in children with undiagnosed and diagnosed asthma. Eur J Pediatr.

[9] Gilson KM, Davis E, Reddihough D (2014). Quality of life in children with cerebral palsy: implications for practice. J Child Neurol.

[10] Felsmann M, Futyma B, Felsmann M (2012). Quality of life in children with epilepsy, evaluated by the parents on the basis of Qolce questionnaire. Medical & Biological Sciences.

[11] Lai JS, Nowinski C, Victorson D (2012). Quality-of-life measures in children with neurological conditions: pediatric neuro-QOL. Neurorehabil Neural Repair.

[12] Rosenberg AR, Orellana L, Ullrich C (2016). Quality of life in children with advanced Cancer: a report from the PediQUEST study. Journal of Pain & Symptom Management.

[13] Wang H, Liu DW, Wang Y (2005). Quality of life and its influencing factors among junior middle school students of the three-gorges reservoir in Chongqing (in Chinese). Modern Preventive Medicine.

[14] Institute of Medicine (2004). Health literacy: a prescription to end confusion.

[15] Nutbeam D (2008). The evolving concept of health literacy. Social Science Medicine.

[16] Guo SJ, Yu XM, Wang L, Sun YY (2013). Factor analysis on health literacy of junior middle school students in China (in Chinese). Chinese Journal of Child Health Care.

[17] Zhuang RS, Li C, Zhu MZ (2013). Status of health literacy and its influencing factors among high school students in Shenzhen city (in Chinese). Chin J Public Health.

[18] Zhang YL, Yu H, Wei WJ, Qiu AM, Chen XL (2012). Current situation of health knowledge and health education among junior middle school students in Xiamen (in Chinese). Chinese Journal of School Health.

[19] Lin PP, Chen ZJ, Zhang J, Fang MZ, Wu X, Xu WL (2013). Hangzhou junior high school students' health literacy: a survey of the status quo and possible countermeasures (in Chinese). Health Research.

[20] Zhang SC, Wan YH, Tao SM (2014). Reliability and structural validity evaluation of chinese youth interactive health literacy questionnaire (in Chinese). Chinese Journal of School Health..

[21] National Health Commission of People's Republic of China. The level of health literacy of Chinese residents in 2012: surveillance results [EB/OL]. Available at: http://www.nhfpc.gov.cn/zhuz/xwfb/201711/308468ad910a42e4bbe9583b48dd733a.shtml. Accessed 20 June 2018.

[22] Burns J, Rapee R (2006). Adolescent mental health literacy: young people's knowledge of depression and help seeking. J Adolesc.

[23] Ghaddar S, Valerio M, Garcia C, Hansen L (2011). Adolescent health literacy: the importance of credible sources for online health information. J Sch Health.

[24] Chang LC (2011). Health literacy, self-reported status and heal promoting behaviours for adolescents in Taiwan. J Clin Nurs.

[25] Cimasi RJ, Sharamitaro AR, Seiler RL (2013). The association between health literacy and preventable hospitalizations in Missouri: implications in an era of reform. J Health Care Finance.

[26] Herman A, Young KD, Espitia D (2009). Impact of a health literacy intervention on pediatric emergency department use. Pediatr Emerg Care.

[27] Lee SYD, Tsai T, Tsai YW (2010). Health literacy, health status, and healthcare utilization of Taiwanese adults: results from a national survey. BMC Public Health.

[28] Yin HS, Mendelsohn AL, Wolf MS (2010). Parents' medication administration errors: role of dosing instruments and health literacy. Archives of Pediatrics & Adolescent Medicine.

[29] Harrington KF, Zhang B, Magruder T (2015). The impact of Parent's health literacy on pediatric asthma outcomes. Pediatric Allergy Immunology & Pulmonology.

[30] Hassan K, Heptulla RA (2010). Glycemic control in pediatric type 1 diabetes:the role of caregiver literacy. Pediatrics.

[31] Sanders LM, Thompson VT, Wilkinson JD (2007). Caregiver health literacy and the use of child health services. Pediatrics.

[32] Morrison AK, Schapira MM, Gorelick MH (2014). Low caregiver health literacy is associated with higher pediatric emergency department use and nonurgent visits. Acad Pediatr.

[33] Song L, Mishel M, Bensen JT (2012). How does health literacy affect quality of life among men with newly diagnosed clinically localized prostate cancer. Cancer.

[34] Wang C, Kane RL, Xu D (2015). Health literacy as a moderator of health-related quality of life responses to chronic disease among Chinese rural women. BMC Womens Health.

[35] González-Chica DA, Mnisi Z, Avery J (2016). Effect of health literacy on quality of life amongst patients with Ischaemic heart disease in Australian general practice. PLoS One.

[36] Wang LY, Wang H, Cheng XT (2016). Development of health literacy scale for middle school students and its reliability and validity analyze(in Chinese). Modern Preventive Medicine.

[37] World Health Organization (1998). Health promotion glossary.

[38] The Ministry of Education of the People's Republic of China (2009). Notice of the ministry of education on the issuance of the outline of health education guidance in primary and secondaryschool students. Bulletin of the state council of the People's Republic of China.

[39] Ministry of public health of the people's Republic of China (2016). Chinese citizens’health literacy-basic knowledge and skills (trial). Chinese Junior of Health Education.

[40] Cheng ZH, Gao BL (1998). The inventory of subjective life quality for child and adolescent: development, reliability, and validity (in Chinese). Chinese Journal of Clinical Psychology.

[41] Wu HR, Liu PL, Meng H (2006). Norm, reliability and validity of children and Adolescents' QOL scale(in Chinese). Chinese Journal of School Health.

[42] Lu YY, Tian Q, Hao YT (2008). Reliability and validity for Chinese version of pediatric quality of life inventory PedsQL4.0 (in Chinese). Journal of Sun Yat-sen University (Medical Sciences).

[43] Chang LC, Hsieh PL, Liu CH (2012). Psychometric evaluation of the Chinese version of short-form test of functional health literacy in adolescents. J Clin Nurs.

[44] Chisolm DJ, Manganello JA, Kelleher KJ (2014). Health literacy, alcohol expectancies, and alcohol use behaviors in teens. Patient Education & Counseling.

[45] Wang CL, Robert LK, Xu DJ (2015). Health literacy as a moderator of health-related quality of life responses to chronic disease among Chinese rural women. BMC Womens Health.

[46] Ye XH, Yang Y, Gao YH (2014). Status and determinants of health literacy among adolescents in Guangdong, China. Asian Pac J Cancer Prev.

[47] Wolf MS, Gazmararian JA, Baker DW (2005). Health literacy and functional health status among older adults. Arch Intern Med.

[48] Bridges SM, Parthasarathy DS, Wong HM (2014). The relationship between caregiver functional oral health literacy and child oral health status. Patient Education & Counseling.

[49] Ownby Raymond L., Acevedo Amarilis, Jacobs Robin J., Caballero Joshua, Waldrop-Valverde Drenna (2014). Quality of life, health status, and health service utilization related to a new measure of health literacy: FLIGHT/VIDAS. Patient Education and Counseling.

[50] Sun AY, Wu YQ, Qian L (2014). Research on health literacy among junior middle school students in Chuxiong (in Chinese). Chin J Sch Health.

[51] Yao Z (2013). Research on knowledge gap and its countermeasures in urban and rural health communication (in Chinese).

[52] Kutner Mark, Greenburg Elizabeth, Jin Ying, Paulsen Christine. The Health Literacy of America's Adults: Results from the 2003 National Assessment of adult literacy. NCES 2006-483. National Center for Education Statistics, 2006, 39(10):685–687.

[53] Vardavas CI, Kondilis BK, Patelarou E, Akrivos PD, Falagas ME (2009). Health literacy and sources of health education among adolescents in Greece. Int J Adolesc Med Health.

[54] Wu RL, Shi HJ, Bei PL (2015). Survey on health literacy among grade seven students in Putuo District of Shanghai in 2012-2013(in Chinese). J Environ Occup Med.

[55] Qiu SY, Zhang JQ, Feng C (2016). Survey on health literacy of junior school students in Hefei (in Chinese). Anhui J Prev Med.

[56] Leng Y, Sun T, Zhang SH (2016). Influence factor analysis on health literacy among city middle school students in Shandong Province (in Chinese). Chinese Journal of Health Education.

